# Association between methylation in nasal epithelial *TSLP* gene and chronic rhinosinusitis with nasal polyps

**DOI:** 10.1186/s13223-019-0389-3

**Published:** 2019-11-21

**Authors:** Jingyun Li, Jian Jiao, Yunbo Gao, Yuan Zhang, Luo Zhang

**Affiliations:** 1 Department of Otolaryngology Head and Neck Surgery, Beijing TongRen Hospital, Capital Medical University, Beijing, 100730 China; 2 Beijing Key Laboratory of Nasal Diseases, Beijing Institute of Otolaryngology, No. 17 HouGouHuTong, DongCheng District, Beijing, 100005 China; 3 Department of Allergy, Beijing TongRen Hospital, Capital Medical University, Beijing, 100176 China

**Keywords:** Chronic rhinosinusitis, DNA methylation, Nasal resistance, Olfactory score, Thymic stromal lymphopoietin (TSLP)

## Abstract

**Background:**

This study was performed to determine whether there was any association between abnormal DNA methylation of a thymic stromal lymphopoietin (TSLP) locus and pathogenesis of chronic rhinosinusitis (CRS).

**Methods:**

A total of 48 CRS patients with nasal polyps (CRSwNP), 28 CRS patients without nasal polyps (CRSsNP) and 21 control subjects were enrolled into the study; and evaluated for serum total IgE level, olfactory score and nasal resistance. Samples were obtained from nasal polyps of CRSwNP patients, ethmoid mucosae of CRSsNP patients and inferior turbinate (IT) mucosa of control subjects during surgery, and used to isolate purified primary human nasal epithelial cells (HNECs). Genomic DNA was extracted from purified primary HNECs of each subject and DNA methylation ratios for a selected region of the TSLP gene were screened the using MassARRAY EpiTYPER.

**Results:**

A total of 17 CpG units were analyzed; of which two CpG units (CpG3 and 22:23:24) had increased methylation ratios in the CRSwNP patients compared to the CRSsNP and control subjects after correction for false discovery rate (FDR) (Q < 0.1). The methylation ratios at both CpG3 and CpG22:23:24 units were positively correlated with olfactory score (r = 0.41, *P *= 0.0001; r = 0.25, *P *= 0.021) and unilateral nasal resistance at 75 Pa (r = 0.24, *P *= 0.04; r = 0.24, *P *= 0.036) and 150 Pa (r = 0.34, *P *= 0.004; r = 0.25, *P *= 0.031). Total nasal resistance at 75 Pa/150 Pa or serum total IgE levels were not correlated with the methylation ratios at either CpG unit.

**Conclusions:**

Increased DNA methylation at the TSLP locus is likely to be associated with CRSwNP pathogenesis; however these findings need to be confirmed in larger multicentre group studies.

## Background

Chronic rhinosinusitis (CRS) is one of the most common inflammatory disorders of the sinus and paranasal sinus mucosa, which affects approximately 5–15% of the general population worldwide [1–3]. CRS can generally be classified into two major phenotypes; including CRS with nasal polyps (CRSwNP) and CRS without nasal polyps (CRSsNP). Although CRS is a highly complex disease, both genetic and environmental factors are believed to contribute to the pathogenesis of this disease.

Epigenetic modification is essential for regulating tissue and stage-specific gene expression during growth and development, and can serve as a mediator of gene-environment interactions in the pathogenesis and progression of disease [4]. DNA methylation is one of the most investigated and important forms of epigenetic modification, and involves the covalent addition of a methyl group to the fifth carbon of a cytosine (C) residue, mostly in the context of CG dinucleotide (CpG). Clusters of these CpG sites (CpG island) are located in the promoter regions of 50% genes [5]. Although hypermethylation of CpG sites at gene promoter regions is commonly thought to be related to transcriptional silencing, some recent studies have demonstrated that hypermethylation of CpG residues was significantly associated with activation of some genes [6, 7]. It has been shown that alteration of DNA methylation may contribute to some mechanisms relevant to CRS pathophysiology, including transcriptional regulation of some T_H_ genes and cytokine production [8].

Thymic stromal lymphopoietin (TSLP) is an IL-7 like and epithelial cell derived cytokine that is commonly recognized as a driver of T_H_2 responses. TSLP stimulates dendritic cells (DCs) to induce naïve CD4^+^ T cell differentiation into T_H_2 cells and cytokine production [9], indicating that TSLP plays an important role in allergic and immune disorders. Indeed, some studies have shown that TSLP may be involved in the aetiology of CRS [10–12]. While one study has demonstrated significant overexpression of TSLP mRNA in NP tissues from CRSwNP patients compared to nasal mucosa from CRSsNP and control subjects [10], another study has shown that the numbers of immunohistochemically stained TSLP^+^ nasal epithelial cells were significantly increased in NP tissues from atopic subjects compared to non-atopic subjects and also strongly correlated with the number of tissue eosinophils and IgE levels in NP tissue [11]. Similarly, studies investigating genetic polymorphisms in the TSLP gene have reported specific single nucleotide polymorphisms (SNPs) to be linked with asthma [13], atopic dermatitis (AD) [14] and allergic rhinitis (AR) [15]. We have previously demonstrated that SNPs in the TSLP gene exhibit a gender and/or nasal polyps-dependent risk for CRS [16].

To our knowledge, the epigenetic change/s in TSLP gene involved in the pathogenesis of CRS is/are presently not well understood. Thus, the aim of the present study was to investigate any relationship between abnormal DNA methylation status of a CpG island in the TSLP gene and pathogenesis of CRS with or without nasal polyps.

## Methods

### Subjects

The study protocol was approved by the Ethics Committee of Beijing Tongren Hospital and all subjects provided written informed consent prior to recruitment. A total of 48 patients with CRSwNP and 28 patients with CRSsNP, fulfilling the criteria of CRS according to the European Position Paper on Rhinosinusitis and Nasal Polyps (EPOS) 2012 guidelines [17] were recruited from the Rhinology Department of Beijing Tongren Hospital, from November 2014 to November 2015. The diagnosis of CRS was confirmed by trained rhinologists, based on patients’ histories, clinical examinations, nasal endoscopies and computed tomographic scans of the sinuses. A total of 21 subjects, who demonstrated no sinonasal disease by rhinoscopy and presented for septoplasty for anatomic variations, were recruited to the study as control subjects.

None of the subjects had used oral or nasal corticosteroids for 4 weeks or antibiotics for 2 weeks before their surgeries. Atopic status was confirmed by detecting IgE antibodies against various common inhalant allergens using the Phadiatop test (Phadia, Uppsala, Sweden). Serum total IgE level was detected using the ImmunoCAP 100 system (Pharmacia, Uppsala, Sweden). Diagnosis of asthma was made by pulmonologists, based on lung function and allergen challenge tests. Nasal polyps score for each subject was evaluated by a different observer using the Davos scoring system. The olfactory function test was performed using the Toyota and Takagi (T&T) olfactometer (Daiichi Pharmaceutical Co. Ltd., Tokyo, Japan), according to the manufacturer’s instructions. The score was assessed as the average of the sum of odor threshold for 5 odorants. For the T&T test, a score of 1 was defined as euosmia, 2 = mild hyposmia, 3 = moderate hyposmia, 4 = severe hyposmia and 5 = anosmia. Nasal resistance was measured at 75 Pa and 150 Pa, respectively, by HRR2 four-phase rhinomanometry (Rhino Lab GmbH, Rendsburg, Germany), as described previously [18]. The left (RL) and right (RR) lateral nasal resistance values were calculated according to Ohm’s law; and the total nasal resistance was calculated using the formula (RL × RR)/(RL + RR), and the unilateral nasal resistance calculated usung the formula (RL + RR)/2.

Tissue samples were obtained from nasal polyps of patients with CRSwNP, ethmoid mucosa of patients with CRSsNP, and inferior turbinate (IT) mucosa of control subjects during surgery. Primary human nasal epithelial cells (HNECs) were derived from these above tissues as previously described [19]. Briefly, the tissue sample was dissociated enzymatically by incubation overnight in medium containing 0.1% protease and the isolated purified epithelial cells were collected by centrifugation at 120 g for 10 min for analysis of DNA methylation.

### DNA methylation analysis by SequenomMassARRAY EpiTYPER

**Fig. 1:**
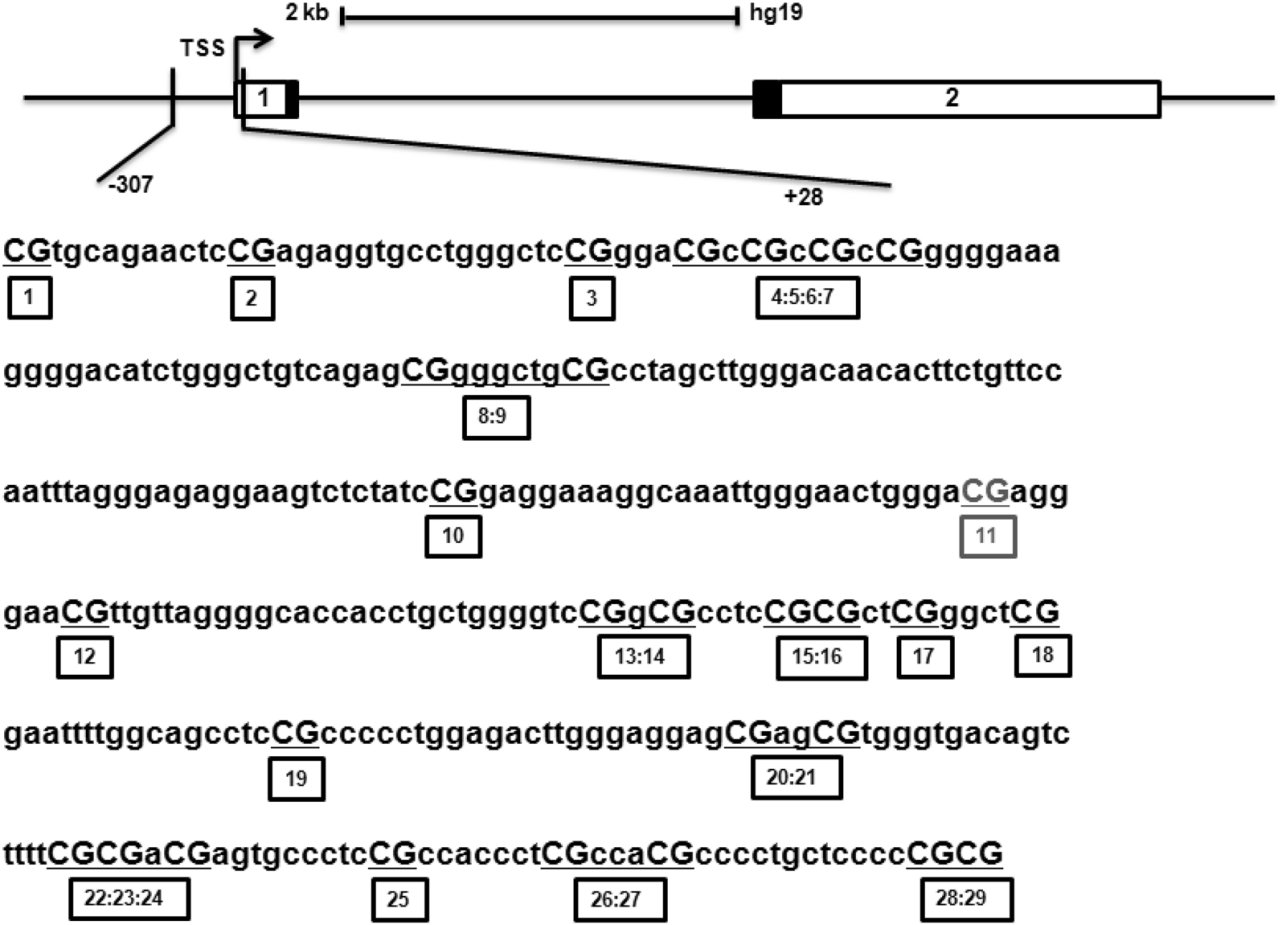
Genomic localization and overview of the CpG sites investigated. In the present study, a large region in a CpG island located in the sfTSLP proximal promoter was covered from − 307 to + 28. CpG11 site could not be measured because of technical limitations of the assay. *TSS* transcription start site

Genomic DNA was extracted from purified primary HNECs of each subject using human tissue DNA kit (Omega Bio-tek, Georgia, CA, USA). DNA quality was assessed by electrophoretic migration on 1% agarose gel, and the DNA concentration was measured by UV spectroscopy at 260 nm. A total of 1 μg genomic DNA was bisulfite-treated with EZ DNA methylation kit (Zymo Research, Orange, CA, USA), and the SequenomMassARRAY EpiTYPER platform (Sequenom, San Diego, CA, USA) was used to detect the DNA methylation ratios for a selected region in each sample, according to the manufacturer’s instructions. The EpiDesigner software (Sequenom, San Diego, CA, USA) was designed to systematically screen the methylation status of 29 CpG sites in the TSLP gene. Due to the cleavage pattern, some CpG sites were analysed in units. The sequence and location of the CpG islands are shown in Fig. 1 and Additional file 1: Figure S1. The positions and sequences of primers used for the EpiTYPER assay to analyze DNA methylation of TSLP locus are shown in Additional file 1: Table S1.

### Statistical analysis

The demographic and clinical characteristics of the study population were expressed as proportions or mean and range, and differences between the three study groups were evaluated by χ^2^ test for categorical variables, one-way ANOVA and Kruskal–Wallis test for continuous variables.

Differences in methylation ratios among CRSsNP, CRSwNP and Control subjects were analysed using nonparametric Kruskal–Wallis test followed by a Mann–Whitney *U* test. The false discovery rate (FDR) was used to correct for multiple testing for the CpG sites; with a Q < 0.1 considered to be significant. Linear regression modelling was used to evaluate the relationship between methylation ratios of associated CpG units and disease status after adjustment for age, gender, smoking, drinking and comorbid asthma and allergic status. Spearman correlation test was used for assessing the presence of any correlation between the methylation status of a CpG unit and clinical factors. SPSS 22.0 software for Windows was used for data analysis. A value of *P *< 0.05 was considered statistically significant.

## Results

### Demographic and clinic characteristics of the subjects

The demographic and clinic characteristics of the subjects in the three study groups are summarized in Table 1. Overall, the three groups were matched for ethnicity and geographical location and had similar ratios of gender, smoking and drinking status, the prevalence of comorbid allergy and asthma, and the mean serum total IgE levels. The two CRS subgroups had significantly older subjects, with higher olfactory scores and total and unilateral nasal resistance, than the control group. A total of 12 patients with CRSwNP (25%) had prior nasal surgeries, compared to none of the control subjects or patients with CRSsNP.

**Table 1:** Demographic and clinical characteristics of study participants

Characteristic	Control subjects	Patients with CRSsNP	Patients with CRSwNP	*P*
No.	21	28	48	
Gender (female/male)	12/9	21/7	35/13	0.34
Age (years), median (range)	44 (18–65)	46.5 (19–68)	56 (18–67)	*0.02*
Allergy, no (%)	1 (4.8)	7 (25)	11 (22.9)	0.15
Asthma, no (%)	0 (0)	0 (0)	5 (10.4)	0.07
Smoking, no (%)	5 (23.8)	7 (25)	18 (37.5)	0.38
Drinking, no (%)	6 (28.6)	7 (25)	17 (35.4)	0.61
Serum total IgE (kU/I), median (range)	43 (2–410)	83.95 (6.04–577)	48 (2.59–1017)	0.38
Polyp score (Davos), median (range)	0	0	3 (1-6)	*< 0.001*
Prior nasal surgeries, no (%)	0 (0)	0 (0)	12 (25.0)	*< 0.001*
Olfactory score, median (range)	1	1 (1–5)	4 (1–5)	*0.003*
Total nasal resistance (Pa/cm^3^/s), 75 Pa, median (range)	0.116 (0–0.301)	0.145 (0.095–0.357)	0.073 (0–8.163)	*0.041*
Unilateral nasal resistance (Pa/cm^3^/s), 75 Pa, mean (range)	0.276 (0.150–0.685)	0.373 (0.200–0.737)	0.463 (0.141–16.811)	*0.001*
Total nasal resistance (Pa/cm^3^/s), 150 Pa, median (range)	0.139 (0–0.354)	0.190 (0–0.441)	0.247 (0–11.768)	*0.045*
Unilateral nasal resistance (Pa/cm^3^/s), 150 Pa, mean (range)	0.303 (0.199–0.797)	0.507 (0.244–2.371)	0.614 (0.121–23.703)	*0.001*

Italic values indicate significance of *P* value (*P* < 0.05)

### DNA methylation in TSLP gene in primary HNECs

TSLP gene is present as two transcript variants coding two distinct isoforms, the long- and short-form TSLP (lfTSLP and sfTSLP, respectively). Based on the CpG island track profiles of UCSC Genome Browser (genome.ucsc.edu), TSLP gene contains one CpG island harboring a total of 29 CpG residues, which locates in the sfTSLP proximal promoter while in the second intron of lfTSLP gene (Fig. 1 and Additional file 1: Figure S1).

**Fig. 2:**
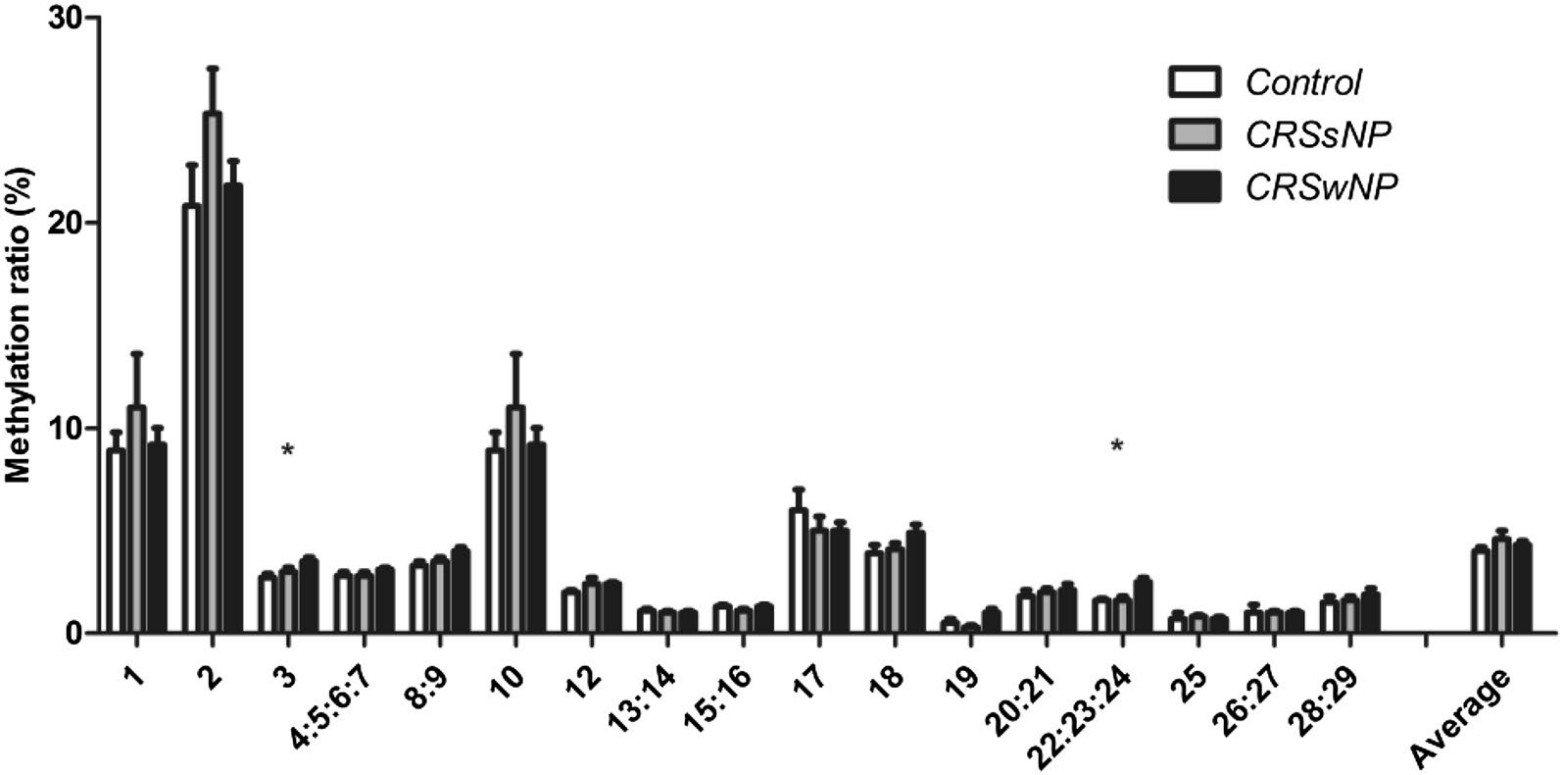
Comparison of methylation ratios at individual CpG residues among CRSsNP and CRSwNP patients and control subjects. Results are expressed as mean ± SEM. FDR was used for multiple testing; with * = Q < 0.1

A total of 17 CpG units were analyzed. As shown in Fig. 2, in the CRSwNP patients, two CpG units (CpG3 and 22:23:24) had increased methylation ratios compared to the CRSsNP and control subjects after correction for FDR (Q < 0.1).

To exclude the effect of confounder factors on the association between methylation ratios of CpG3 and CpG22:23:24 units and CRSwNP, we performed linear regression models and found that these significant relationships remained robust even after adjusting for potential confounders including age, gender, smoking, drinking and comorbid asthma and allergic status (Table 2). However, greater age was significantly associated with increased methylation ratios of CpG3, but not with increased methylation ratios of CpG22:23:24 in the *TSLP* promoter (Table 2).

**Table 2:** Multivariate analysis of factors influencing methylation ratios at CpG3 and CpG22:23:24 sites within *TSLP* promoter

Factors	Standardized beta	95% CI	*P*
CpG3			
Disease status	0.26	0.001–0.006	*0.019*
Gender	− 0.11	− 0.008–0.003	0.38
Age	0.25	2.6E−5–3.8E−4	*0.025*
Smoking	− 0.13	− 0.009–0.003	0.32
Drinking	− 0.01	− 0.006–0.006	0.92
Comorbid asthma	0.08	− 0.007–0.014	0.47
Allergic disease	− 0.03	− 0.006–0.005	0.77
CpG22:23:24			
Disease status	0.31	0.002–0.009	*0.006*
Gender	0.04	− 0.006–0.009	0.73
Age	0.08	− 1.5E−4–3.3E−4	0.48
Smoking	− 0.16	− 0.01–0.003	0.23
Drinking	0.15	− 0.004–0.013	0.27
Comorbid asthma	− 0.17	− 0.02–0.002	0.10
Allergic disease	− 0.04	− 0.009–0.006	0.72

Italic values indicate significance of *P* value (*P* < 0.05)

### Impact of DNA methylation in TSLP gene on clinical factors

Both methylation ratios at CpG3 and CpG22:23:24 units were positively correlated with olfactory score (r = 0.41, *P *= 0.0001; r = 0.25, *P *= 0.021) as well as unilateral nasal resistance at 75 Pa (r = 0.24, *P *= 0.04; r = 0.24, *P *= 0.036) and 150 Pa (r = 0.34, *P *= 0.004; r = 0.25, *P *= 0.031), but not correlated with total nasal resistance at 75 Pa or 150 Pa, nor serum total IgE levels (Table 3).

**Table 3:** Correlation between methylation ratios at CpG3 and CpG22:23:24 sites within *TSLP* promoter and demographic parameters

Characteristic	CpG3		CpG22:23:24	
	r	*P*	r	*P*
Serum total IgE	0.16	0.23	− 0.042	0.75
Olfactory score	0.41	0.0001	0.25	0.021
Total nasal resistance, 75 Pa	0.095	0.416	0.13	0.26
Unilateral nasal resistance, 75 Pa	0.24	0.04	0.24	0.036
Total nasal resistance, 150 Pa	0.17	0.16	0.13	0.28
Unilateral nasal resistance, 150 Pa	0.34	0.004	0.25	0.031

## Discussion

Most of the studies on the role of TSLP in the pathogenesis of CRS to date have focused on non-epigenetic and genetic mechanisms; with relatively little known on the epigenetic mechanisms involved. The present study employed MassARRAY EpiTYPER technique to widely screen the methylation status of a CpG island in the TSLP gene and then evaluated any associations between the methylation ratios of these CpG units and CRS. We found that the methylation ratios of two CpG units, CpG3 and CpG22:23:24 were strongly associated with susceptibility to CRSwNP. Furthermore, analysis of clinical symptoms showed positive correlations between the methylation ratios of CpG3 and CpG22:23:24 and olfactory score and unilateral nasal resistance, respectively. Indeed, it is possible that the association the methylation ratios of CpG3 and CpG22:23:24 and olfactory score may partly be age-related because greater age was significantly associated with increased methylation ratio of particularly CpG3 and because olfactory function declines with age.

TSLP has pleiotropic biological and pathological functions;with overexpression of TSLP detected in several allergic diseases, including asthma, AD, AR and food allergy [20]. In the thymus, TSLP is expressed by Hassal corpuscles and regulates the capacity of dendritic cells(DCs) and plasmacytoid DCs to promote the differentiation of natural regulatory T cells [21, 22]. Indeed, one study demonstrated that TSLP-activated DCs induced a robust homeostatic polyclonal expansion of T cells [23]. Moreover, TSLP has been shown to induce a strong T_H_2-mediated inflammation by increased production of numerous T_H_2 cytokines; such as IL-4, IL-5 and IL-13 as well as TNF-α [9], through upregulation of OX40 ligand expression on TSLP-activated DCs [24]. Similarly, TSLP levels have been shown to be significantly increased in CRSwNP nasal mucosa compared to CRSsNP and control nasal mucosa; and furthermore positively associated with markers of T_H_2 responses and eosinophilia [10–12]. These findings suggest that elevated levels of TSLP may be involved in the pathogenesis of CRSwNP through the regulation of T_H_2 inflammatory cytokine accumulation. A genetic study from our laboratory has previously shown that the TSLP gene may be important in conferring susceptibility to CRS, especially CRSwNP [16]. While some polymorphisms in or near the TSLP gene have been reported to be associated with multiple allergic diseases [13–15], the specific mechanism underlying the involvement of these polymorphisms has yet to be revealed. Thus, the present study was undertaken with the view that investigation of methylation status of the TSLP gene might provide better understanding of a role of TSLP gene in CRS.

TSLP has two distinct isoforms (long and short) which are encoded by two different transcript variants [25]. The canonical variant encodes the longer isoform (known as lfTSLP), which is the type thought to promote allergic inflammation in diseases. Similarly, the transcript variant 2 encodes the shorter isoform (known as sfTSLP), which is reported to play an antimicrobial role [26]. Data on gene structure of human TSLP transcripts in the UCSC Genome Browser shows that there are two putative promoter regions to activate the two TSLP transcript variants. The ENCODE track profiles show that the short isoform promoter, which covers the CpG island investigated in the present study, has a high capacity to bind multiple different transcription factors. It is possible that methylation at specific CpG sites may modify these sites and therefore influence the affinity for transcription factors near the cis-elements. In the present study we found that overall methylation ratios at the sfTSLP promoter were not significantly different between HNECs of CRSsNP and CRSwNP patients. In contrast, a study by Luo and colleagues [27] has reported that average methylation at the remote promoter of lfTSLP was significantly decreased in skin lesions from patients with AD compared with healthy controls. It is possible that the discordance between our study and that of Luo and colleagues may be due to differences in the CpG sites assessed for in the promoter regions of sfTSLP and lfTSLP, and differences in the cell types investigated. However, comparison of the difference in methylation ratios at individual CpG sites or units among CRSwNP, CRSsNP or controls in the present study has demonstrated that there was significantly increased of DNA methylation at the CpG3 and CpG22:23:24 sites in CRSwNP patients. While the precise role of hypermethylation of the CpG3 and CpG22:23:24 sites is presently not clear, it is possible that CpG3 and CpG22:23:24 hypermethylation may act as a positive regulatory mechanism of lfTSLP; particularly as CHIP-seq experiments from ENCODE track have exhibited binding of some cis-acting transcriptional repressors such as CTBP2 and CTCF in this region. Thus, hypermethylation at CpG3 and CpG22:23:24 may be a mechanism to disrupt binding of these transcriptional repressors to these sites. On the other hand, it is possible that CpG3 and CpG22:23:24 hypermethylation may involve the regulation of sfTSLP; particularly as sfTSLP has an opposite immune function compared with lfTSLP. This may be important as upregulation of sfTSLP is one of the most important mechanisms of vitamin D-mediated protection against the airway inflammation [25, 28]. Recently, Lan and colleagues [29] reported that sfTSLP was decreased in CRSwNP tissue after *S. aureus* infection, suggesting sfTSLP may participate in the nasal mucosal defence against bacterial infection. In the present study, the effect of several potential confounders; including age, gender, smoking, drinking and comorbid asthma and allergic status were also taken into account, and after adjusting for these factors, hypermethylation at CpG3 and CpG22:23:24 was still found to be associated with CRSwNP. Although our results suggest that the methylation status of TSLP may be a key independent factor and implicate hypermethylation of TSLP DNA in increased susceptibility to CRSwNP, presently these findings do not provide any clear indications with regard to the specific mechanism/s involved in this aspect.

Sensorineural olfactory loss may lead to the hyposmia in CRS patients. Local increase of inflammatory cytokines such as TNF-α and INF-γ can suppress the olfactory neuron function, turnover and survival [30–32]. Similarly, TSLP also have neurotoxic potential, and the epithelial cells in the airway can directly communicate to cutaneous sensory neurons via TSLP to promote itch [33]. Thus, it is conceivable that TSLP may be modulated in olfactory dysfunction in CRS patients. Indeed, the finding from the present study that the methylation ratios of CpG3 and CpG22:23:24 were positively associated with olfactory score provide a pointer for further investigation to test this hypothesis. Similarly, our finding that hypermethylation at CpG3 and CpG22:23:24 was highly correlated with unilateral nasal resistance rather than total nasal resistance raises the possibility that TSLP may also be involved in the aetiology of nasal obstruction, which is one of the most common symptoms in CRS patients. Currently, nasal airflow resistance is frequently used for the objective evaluation of rhinostenosis [34]. The results in the present study provided evidence that hypermethylation at CpG3 and CpG22:23:24 might have effects on clinical phenotypes, including hyposmia and nasal obstruction, in the pathophysiology of CRS.

However, the findings of this study are slightly limited, particularly in view of differences in some baseline demographic characteristics of subjects in the CRSwNP group compared to the CRSsNP and control groups. Thus, given that the baseline demographic for age was statistically significantly higher in patients with CRSwNP than in control and CRSsNP patients; and multivariate analysis also demonstrated that, apart from the disease status, only age significantly influenced the methylation ratio of specifically CpG3 site within the *TSLP* promoter, it is possible that the observed effects on methylation within the *TSLP* promoter may at least partly be age-related. Indeed, it is likely that the significant difference noted for the baseline demographic of higher olfactory scores in patients with CRSwNP than in control and CRSsNP patients, was also age-related, because olfactory function declines with age. Furthermore, this study is also somewhat limited by the relatively small sample size, and therefore these findings need to be validated in larger age-matched cohorts. Moreover, the levels of TSLP protein and receptor in nasal mucosa or nasal secretions were not measured, as increased levels of these proteins in CRSwNP patients would have provided some support for the present findings for the relationship between methylation status of TSLP and CRSwNP.

## Conclusion

In conclusion, this preliminary study provides new insight into the role of TSLP in the development of CRS and shows that increased DNA methylation at the TSLP locus is likely to be associated with CRSwNP pathogenesis; however these findings need to be confirmed in multicentre studies with larger age-matched cohorts. Further, although bioinformatics databases predict that CpG3 and CpG22:23:24 units can disrupt binding of several transcriptional factors, experimental evidence of the functional effects of methylation in these sites is still lacking. Nevertheless, this approach may be useful for further investigation of the aetiology of CRS to fill the gap of genetic and non-epigenetic mechanisms.

## Supplementary information

**Additional file 1: Table S1.** Positions and sequences of primers used for the EpiTYPER assay to analyze DNA methylation of TSLP locus. **Figure S1.** Schematic representation of the human TSLP locus on UCSC Genome Browser (hg19).

## Data Availability

We would like to provide the raw data to support the information presented in this publication.

## Abbreviations

AR: allergic rhinitis
AD: atopic dermatitis
CRS: chronic rhinosinusitis
CRSwNP: CRS with nasal polyps
CRSsNP: CRS without nasal polyps
DCs: dendritic cells
FDR: false discovery rate
HNECs: human nasal epithelial cells
IT: inferior turbinate
SNPs: single nucleotide polymorphisms
TSLP: thymic stromal lymphopoietin
