# Supplementary material for: Association between methylation in nasal epithelial TSLP gene and chronic rhinosinusitis with nasal polyps
Source: Allergy Asthma Clin Immunol. 2019 Nov 21;15:71. doi: 10.1186/s13223-019-0389-3 (PMC6873565; doi:10.1186/s13223-019-0389-3)
Supplement: Supplementary file 1 — Additional file 1: Table S1. Positions and sequences of primers used for the EpiTYPER assay to analyze DNA methylation of TSLP locus. Figure S1. Schematic representation of the human TSLP locus on UCSC Genome Browser (hg19). [file 13223_2019_389_MOESM1_ESM.doc]

**Additional file 1: Table S1.** Positions and sequences of primers used for the EpiTYPER assay to analyze DNA methylation of TSLP locus.

| Assay | Position | Direction | | Sequence (5’ to 3’) |
| --- | --- | --- | --- | --- |
| 1 | -363 to -102 | Reverse | aggaagagagTAGTGTTGTTGGTTTTTTTTAGGGA | |
|  |  | Forward | cagtaatacgactcactatagggagaaggctAACCCCAACAAATAATACCCCT | |
| 2 | -125 to +63 | Reverse | aggaagagagTTAGGGTTGAGTAGAGTAAGGAAGAATTA | |
|  |  | Forward | cagtaatacgactcactatagggagaaggctTTAAAAACACCACCTACTAAAATCC | |

Lower case denotes sequence tags necessary for the EpiTYPER protocol.

**Additional file 1: FIGURE S1.** Schematic representation of the human TSLP locus on UCSC Genome Browser (hg19).

**
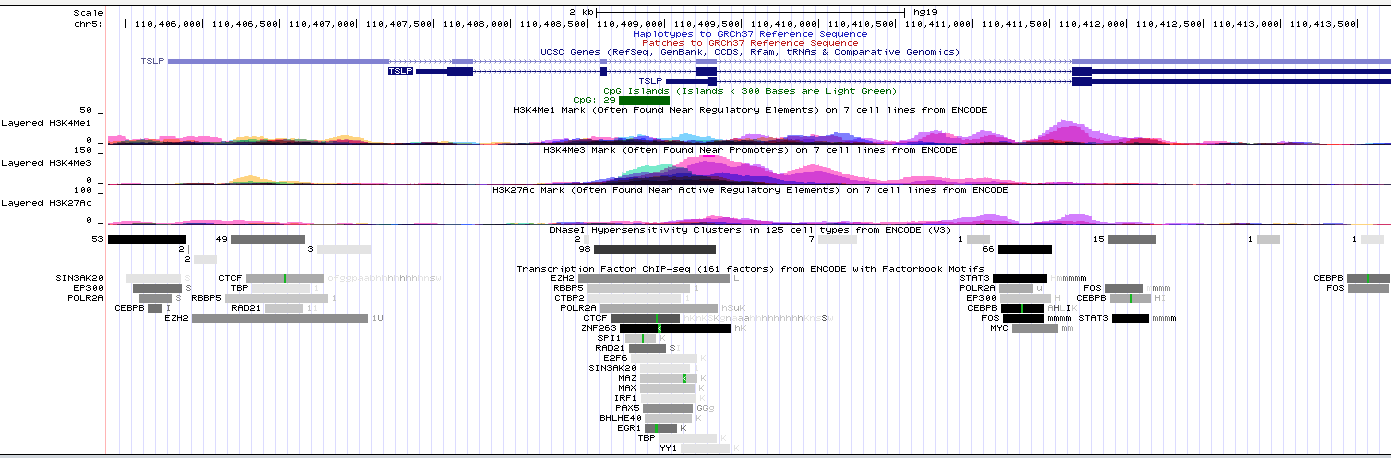
**
